# Cytogenetic characterization of *Hoplias malabaricus* (Bloch, 1794) from the Ctalamochita River (Córdoba, Argentina): first evidence for southernmost populations of this species complex and comments on its biogeography

**DOI:** 10.3897/CompCytogen.v11i1.10262

**Published:** 2017-01-09

**Authors:** Diego Javier Grassi, Ana Claudia Swarça, Jorge Abdala Dergam, María Cristina Pastori, Alberto Sergio Fenocchio

**Affiliations:** 1 Department of Molecular Therapeutics, The Scripps Research Institute. 130 Scripps Way. 33458, Jupiter, Florida, United States of America; 2 Departamento de Histologia, CCB, Universidade Estadual de Londrina. 86051-970, Caixa Postal 6001, Londrina, Paraná, Brazil; 3 Departamento de Biologia Animal, Campus Universitario, Universidade Federal de Viçosa, 36570-000, Minas Gerais, Brazil; 4 Departamento de Genética, Universidad Nacional de Misiones. Instituto de Biología Subtropical (IBS UNaM-CONICET). Félix de Azara 1552. 3300, Posadas, Misiones, Argentina

**Keywords:** Biogeography, cytogenetics, Hoplias malabaricus, karyomorph A

## Abstract

*Hoplias malabaricus* (Bloch, 1794), a predatory freshwater fish with a wide distribution throughout South America, represents a species complex with seven well characterized karyomorphs at the cytogenetic level. Although this species has been extensively studied in several Brazilian basins, data are still scarce for hydrographic systems from other South American countries. This study aims to characterize cytogenetically the *Hoplias malabaricus* populations from the Argentinean Central Region, close to the southernmost distribution of this species complex. A total of 32 specimens from the Ctalamochita River, a tributary of Lower Paraná Basin located in the province of Córdoba, were analyzed using cytogenetic techniques (Giemsa staining, C- and Ag-NOR banding and fluorescent in situ hybridization with 18S rDNA). All the specimens showed diploid number 2n=42, chromosomic formula 22m + 20sm and absence of sexual chromosomes. Thus, the analyzed populations belong to the karyomorph named A. These populations showed a remarkable degree of divergence in their cytogenetic traits such as karyotypic formula, C-banding, NORs and 18S rDNA patterns for *Hoplias malabaricus* from other populations bearing the same karyomorph in the Middle and Upper Paraná Basin. These findings are consistent with molecular data from a recent study (where specimens collected in the present work were included), which indicate a closer phylogenetic relationship of *Hoplias malabaricus* populations from the Ctalamochita River with those from the Uruguay basin and the coastal regions of South Brazil than with populations from the Middle and Upper Paraná Basin. Overall, these pieces of evidence highlight the distinctive features of *Hoplias malabaricus* from the Ctalamochita River, and also reveal a complex history of dispersion of these populations. The present work is the first to provide cytogenetic information and include some phylogeographic aspects of *Hoplias malabaricus* populations living in close proximity to the southernmost extreme of its distribution area. Therefore, this study expands significantly upon the previously known geographical coverage for karyomorph A and contributes to a better understanding of the karyotypic diversification within this species complex.

## Introduction

*Hoplias malabaricus* (Bloch, 1794) is a predatory freshwater fish that belongs to the order Characiformes and family Erythrinidae. It is a widespread species inhabiting 44 out of 52 South American ecoregions proposed by Abell et al. (2008), in most of the hydrographic basins of South America (Oyakawa 2003).

Classically, *Hoplias malabaricus* specimens have been recognized by a few morphological features, such as convergence of dentary bone toward the symphysis, resulting in a ‘‘V’’ shape pattern and tooth plates in the tongue. Although *Hoplias malabaricus* has been considered as a single biological species in reference to its morphological traits, every sampled population shows a bimodal pattern of variation: they are either 2n=42 or 2n=40 with slight modifications. Several cytogenetic studies indicate that *Hoplias malabaricus* represents a species complex, with seven well characterized karyomorphs (or karyotypic variants, also known as cytotypes) nominated with letters A to G, which differ with regards to their diploid numbers, chromosome morphology and the presence of sex chromosome systems (Bertollo et al. 1983, 1997a, 1997b, Dergam and Bertollo 1990, Bertollo and Mestriner 1998, Born and Bertollo 2000, 2001). Some karyomorphs as A, C and F have a wide geographic distribution throughout South America, whereas others are either endemic or restricted to specific drainages in Brazil (Bertollo et al. 2000, Da Rosa et al. 2014). In some cases, two and even more karyomorphs have been found coexisting in sympatric conditions without detection of hybrid forms (Lopes and Fenocchio 1994, Scavonne et al. 1994, Lopes et al. 1998, Lemos et al. 2002, Pazza and Júlio 2003, Born and Bertollo 2006, Da Rosa et al. 2009a, 2009b, 2010).

Frequently, cytogenetic evidence is also supported by, and consistent with, molecular data obtained from analysis of nuclear and mitochondrial DNA sequences (nuDNA and mtDNA), which reveal a marked divergence and genetic structuration between karyomorphs, suggesting reproductive isolation between the karyomorphs. Additionally, combination of cytogenetic and molecular approaches together with geological information (i.e., estimate data of ancient episodes such as stream piracy between rivers, orogenic activity, etc., involved in vicariance events) has been particularly useful in the study of dispersal events and phylogenetic relationships (Dergam et al. 1998, 2002, Santos et al. 2009, Jacobina et al. 2011, Pereira et al. 2013).

All the aforementioned evidence strongly indicates the existence of a complex of cryptic species grouped into the typical *Hoplias malabaricus* morphotype (Bertollo et al. 1986, 2000), and also shows the relevance of these organisms as an exceptional model for studying karyotypic evolution and biogeography.

It is noteworthy to mention that, although the lack of detailed morphometric information has led to group different species, well defined at the genetic level, in a single one on the basis of a common morphology, some karyomorphs of *Hoplias malabaricus* seem to exhibit subtle morphological differences. For example, in a recent study focused on analysis of morphometric parameters (Azpelicueta et al. 2015) a new species of the genus *Hoplias* has been described. This species inhabiting the lower Paraná River (Misiones, Argentina) has been named *Hoplias mbigua*. However, has been suggested that *Hoplias mbigua* would actually be the karyomorph C of *Hoplias malabaricus* previously described in that region of Paraná Basin.

Despite its wide geographic distribution, most of karyotypic and molecular research in *Hoplias malabaricus* populations has been carried out in Brazil and the few studies conducted in Argentina have been restricted to Mesopotamic region (Lopez and Fenocchio 1994, Bertollo 1996, Dergam et al. 1998, Lopes et al. 1998). In this context, the central region of Argentina, also called Pampa Plain, has never been examined before. Taking into consideration that the ichthyofauna of the Pampa Plain presents the southernmost distribution range of many Neotropical species, increasing the geographical coverage may lead to a better understanding of karyotypic diversification and the historical biogeography of *Hoplias malabaricus* and the regional fauna of the Pampa Plain.

The Pampa Plain is a vast region characterized by gentle slopes, occasionally interrupted by low geomorphological reliefs. The hydrographic systems developed in this area form both endorheic and exorheic basins. Among the latter, the Ctalamochita River (also known as Tercero River) is one of the most important hydrographical systems. This river, located in the province of Córdoba, is a tributary of the Lower Paraná River Basin. Along the Ctalamochita River, there are five reservoirs (artificial lakes), the two most important of them called Embalse Río Tercero (built in 1936) and Piedras Moras (built in 1978). The fish assemblage in the Ctalamochita River is characterized by the presence of members of the families Characidae, Pimelodidae and Erythrinidae, among others. Therefore, *Hoplias malabaricus* is an indigenous species (Haro et al. 1996, Bistoni and Hued 2002).

The aim of the present study was characterizing at the cytogenetic level *Hoplias malabaricus* populations from headwater of the Ctalamochita River using standard and fluorescent in situ hybridization techniques.

## Material and methods

**Figure 1.**
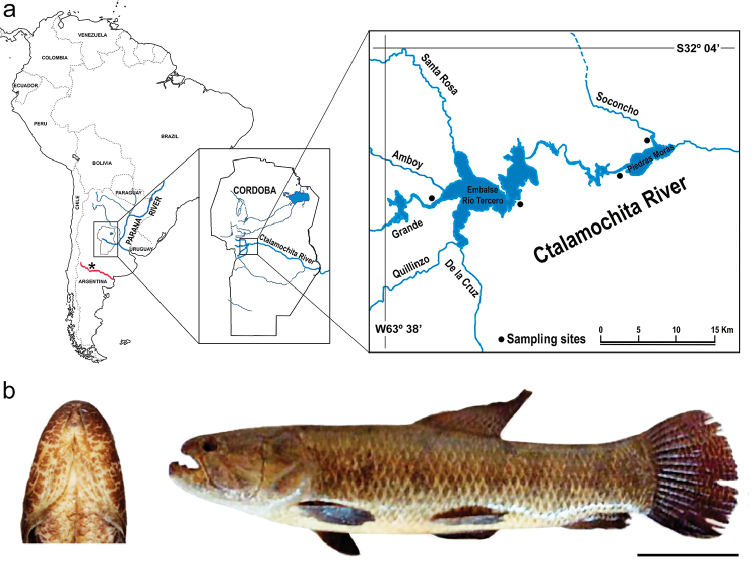
**a** Ctalamochita River location in Province of Córdoba, Argentina (left and middle images). Asterisk (*) indicate location of the Colorado River in Argentina (in red), the limit of distribution of *Hoplias malabaricus* and most of Neotropical fishes. Distribution of sampling sites is indicated along headwater basin (right image, black dots) **b** Representative specimen of *Hoplias malabaricus* caught in the Ctalamochita River and analyzed in the present study (right picture). All specimens showed the typical morphological feature identifying *Hoplias malabaricus*, the V-shaped gular region (left picture). Bar = 10 cm.

Thirty two specimens of *Hoplias malabaricus* consisting of 8 males, 10 females and 14 specimens of undetermined sex were collected in the headwaters of the Ctalamochita River, Córdoba Province, Argentina (Fig. 1a) during December 2004 to March 2005. In order to study the Ctalamochita River headwaters and some of its tributaries, four different sampling sites in the two main reservoirs were chosen (Fig. 1a, black dots, listed from left to right):

- Embalse Río Tercero reservoir and Rio Grande stream

# 1 (32°13'S, 64°32'W): 4 specimens (2 males, 2 females)

# 2 (32°13'S, 64°25'W): 14 specimens (14 juvenile stages, undetermined sex)

- Piedras Moras reservoir and Soconcho stream

# 3 (32°11'S, 64°19'W): 3 specimens (1 male, 2 females)

# 4 (32°11'S, 64°18'W): 11 specimens (5 males, 6 females)

Specimens (Fig. 1b, right) were identified according to Oyakawa (1990). In all cases the rami of the dentary bone were convergent toward the symphysis, resulting in a ‘‘V’’ shape pattern (Fig. 1b, left).

Mitotic chromosome preparations were obtained according to the technique described by Foresti et al. (1993). The method described by Kligerman and Bloom (1977) was used for the analysis of meiotic chromosomes from male gonads.

Nucleolus organizer regions (Ag-NORs) were visualized with silver staining following Howell and Black (1980). Assays conducted to reveal the pattern of constitutive heterochromatin (C banding) were carried out according to Sumner (1972).

Fluorescent *in situ* hybridization (FISH) experiments were performed using biotinylated 18S rDNA probes (1700 pb long fragments) obtained from the nuclear DNA of the fish *Oreochromis niloticus* (Linnaeus, 1758) labeled with biotin-14-dATP by nick translation (Gibco cat N°18247-015), according to the manufacturer’s instructions. The hybridization technique, post-hybridization washes and visualization were carried out following Swarça et al. (2001).

The preparations were analyzed with an Olympus BX50 microscope, and the best metaphases were captured with a SONY camera, model Exware HAD coupled to the microscope. The FISH slides were observed and the images acquired with a Leica DM 4500 microscope equipped with a DFC 300F9 camera and Leica IM50 4.0 software.

More than thirty metaphases from each specimen were analyzed and the best of them were used to make karyotypes. The chromosomes were arranged in groups classified according to their arm ratios as metacentrics and submetacentrics (Levan et al. 1964, Guerra 1986).

Tissue samples from several specimens were collected for further molecular analysis (study of sequences of mitochondrial and nuclear DNA) and were deposited in the Laboratory of Molecular Systematic Beagle, Universidade Federal of Viçosa, Minas Gerais, Brazil.

The entire collection was split in two groups, which were deposited at the Museum of Zoology, Universidad Nacional of Córdoba, Argentina (specimens numbered from 1 to 18) and at Fish Cytogenetics Laboratory, Universidad Nacional of Misiones, Argentina (specimens numbered from 19 to 32).

## Results

**Figure 2.**
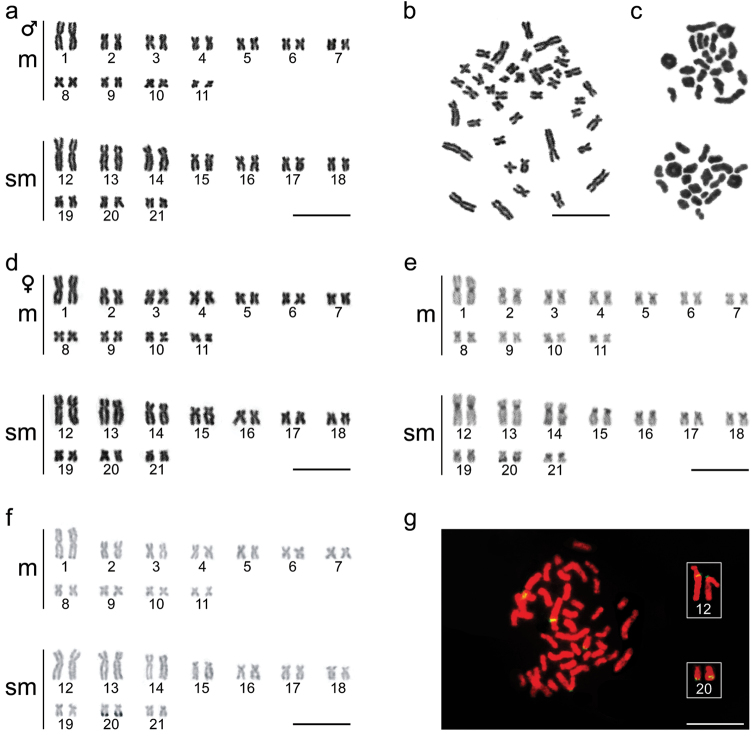
**a** Male *Hoplias malabaricus* karyotype with conventional Giemsa staining (karyomorph A) **b** Metaphase corresponding to karyotype showed in (**a**) **c** Male meiotic metaphase showing formation of 21 bivalents **d** Female *Hoplias malabaricus* karyotype (karyomorph A) **e** C-banding karyotype exhibiting centromeric staining in most of chromosomes and telomeric signal in pairs number 14 and 20 **f** Ag-NOR banding karyotype displays telomeric signal in chromosome pair 20 **g** FISH with 18S rDNA probes labeling two chromosomic pairs, numbers 12 and 20 (boxed). Bar = 5 µm.

All the specimens of *Hoplias malabaricus* collected from the headwater region of the Ctalamochita River exhibited a diploid number of 2n=42 chromosomes. The karyotype was composed of 22m + 20sm, with a NF=84 (Fig. 2a, b and d).

Analysis of meiotic preparations allowed the identification of 21 bivalents without atypical pairing among them, supporting lack of chromosomal differentiation between sexes (Fig. 2c).

Patterns of heterochromatin distribution revealed by C-banding were mostly associated with centromeric regions of all chromosomes of the complement, as well as in the telomeric region of some pairs (Fig. 2e). Metacentric pair 1 and submetacentric pair 12 showed a conspicuous band in centromeric/pericentromeric position. Additionally, submetacentric pairs 14 and 20 exhibited big heterochromatin blocks in telomeric positions in the long arms, but in the former case the signal was weak and diffuse.

Nucleolus Organizer Regions identified by impregnation by silver nitrate (AgNORs) were located in telomeric position on the long arm of a small chromosome pair number 20 (Fig. 2f). In some cases, weak NOR-positive bands were observed in pericentromeric position of submetacentric pair number 12 (data not shown).

These data coincided with results from FISH technique with 18S rDNA probes, which revealed two positive submetacentric pairs: pair number 12, clearly identified due to its size and exhibiting probe signal in its pericentromeric position, and pair number 20, showing probe signal in its long arm (Fig. 2g).

**Figure 3.**
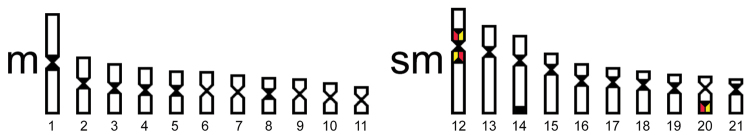
Idiogram referring to the *Hoplias malabaricus* populations (karyomorph A) from the Ctalamochita River, highlighting the chromosome markers. Black: C-positive heterochromatin; red: 18S rDNA sites; yellow: nucleolar organizer regions (NORs). Putative pericentromeric NOR in chromosome pair 12 was also included.

It is important to note that, concerning all chromosome markers and macrostructural features analyzed in this study, no significant differences were found between populations of Embalse Río Tercero and Piedras Moras reservoir. Thus, it seems these populations are relatively homogeneous (Fig. 3).

## Discussion

*Hoplias malabaricus* populations from the headwater of the Ctalamochita River (Córdoba Province, Argentina) were characterized at the cytogenetic level, providing the first data for this hydrographic system and also for the Argentinean Central Region. These populations exhibited karyotypes with 2n=42 chromosomes composed of 11 metacentric and 10 submetacentric pairs, without apparent heteromorphic sex chromosomes. These results qualify them as belonging to karyomorph A previously described by Bertollo et al. (2000).

Despite sharing some chromosomal similarities with other karyomorph A-bearing populations occurring in the Upper and Middle regions of Paraná Basin, cytogenetic traits of the Ctalamochita population such as karyotypic formula, C-banding, NORs and 18S rDNA patterns were significantly divergent (Figure 3). For example, in the case of *Hoplias malabaricus* populations from Pântano Stream and Cuiabá River described by Cioffi et al. (2009), they exhibited karyotypic formulae 22m + 20sm and multiple and bitelomeric NORs and 18S rDNA sites, located on chromosome pairs 5, 16, 18 and 21. Additionally, in all *Hoplias malabaricus* populations from Tibagi, Ivaí and Iguaçu Rivers described by Vicari et al. (2005) their karyotypes were composed of 24m + 18sm with multiple and bitelomeric NORs and 18S rDNA sites, located on chromosome pairs 10, 16 and 21. In a recent work, Gemi et al. (2014) analyzed *Hoplias malabaricus* populations from Iguaçu River, whose karyotypes were composed of 24m + 18sm with multiple and bitelomeric NORs located on chromosome pairs 7 and 10. Ribosomal 18S rDNA probes marked chromosome pairs 4, 7, 10, 13, 16, 20 and 21.

Fixation of chromosome rearrangements within major karyomorphs is not completely surprising taking into consideration some biological aspects of *Hoplias malabaricus*, as its wide distribution in most of South America basins, sedentary habits and adaptations to live in small and isolated populations. These features would favor a random fixation of chromosome rearrangements and, thus, certain degree of intra-karyomorph variation would be expected (Sites and Moritz 1987). Indeed, extensive karyotypic variation in karyomorph A has been reported for populations living in adjacent basins (Born and Bertollo 2001, Vicari et al. 2005, Cioffi et al. 2009, Blanco et al. 2010, Da Rosa et al. 2014). In this regards, populations from the Ctalamochita River seem to follow the rule since they display a unique character combination, diverging from other populations of the A karyomorph inhabiting the Paraná Basin and other hydrographical systems.

With respect to distinctive cytogenetic features of these populations, it is important to highlight relevant traits for submetacentric chromosome pairs 12 and 20. In both cases the presence of heterochromatic bands coincident with 18S rDNA sites was demonstrated; in chromosome pair 12 located in pericentromeric position and in chromosome pair 20 in the long arm. With regards to Ag-NORs only chromosome pair 20 had a clear, positive staining for silver nitrate impregnation, showing a conspicuous positive NOR in telomeric position of the long arm. As was mentioned previously, in some cases (approximately 5%) weak NOR-positive staining was observed in pericentromeric position of submetacentric pair number 12, but it was not possible conclude if this variability was caused by limited accessibility of silver nitrate to highly compacted heterochromatic regions or by the occurrence of structural Ag-NOR polymorphisms (Born and Bertollo 2000, Vicari et al. 2005).

Overlapping of C- and Ag-NOR banding suggests the presence of heterochromatic blocks interspersed with ribosomal cistrons in the same region, as previously reported for several chromosomic pairs in karyomorph A (Vicari et al. 2003, 2005, Blanco et al. 2010, Cioffi et al. 2010). A possible role for these NOR / heterochromatin associations in the evolution of sex chromosomes in other *Hoplias malabaricus* karyomorphs has been advanced by Vicari et al. (2003). In fact, recent data indicate that the differentiation of sexual chromosomal pair in karyomorph B, which is clearly derived from the sympatric karyomorph A, could have occurred by accumulation of heterochromatin and 18S rDNA cistrons in the long arms of the submetacentric chromosome pair 21 of some Brazilian populations belonging to karyomorph A (Cioffi et al. 2010, 2011).

These high levels of chromosomal differentiation within the 2n=42 A karyomorphs proposed by Bertollo et al. (2000) has received recent molecular support in a supertree obtained with the ATPAse6 gene (in which specimens collected in the present work were included), where the Ctalamochita populations seem phylogenetically most closely related to 2n=42 A karyomorphs from left bank tributaries of the Uruguay River and populations from the headwaters of the Uruguay River, and relatively closely to other coastal populations from South of Brazil. In contrast, 2n=42 A karyomorphs of the Upper and Middle Paraná are included in other, distant haplogroups (Santos 2013). Additionally, according to this analysis, it is possible to postulate a direction of dispersal originating from southern coastal basins of Brazil to the Uruguay River and the Ctalamochita hydrographic system.

Although the spread of karyomorph A populations through interaction between coastal drainages and dispersal into continental basins has been previously studied and demonstrated only for restricted Brazilian coastal regions (Dergam et al. 2002, Santos et al. 2009), a possible scenario for larger scale dispersion events involving other, unrelated obligatory freshwater fishes is plausible. In fact, a recent phylogeographic study including four genera of the family Loricariidae suggested that current geographical distribution of these genera is supported by dispersion events from the southern coastal rivers of Brazil to the Uruguay River and from the Uruguay River to the De la Plata River, through stream piracy from coastal Brazilian regions and drainage rearrangements of Paraná basin (Cardoso 2013).

Additionally, fluctuations of sea level during glacial-interglacial cycles and temporary connections between adjacent basins would also have had an important role in spreading karyomorph A *Hoplias malabaricus* populations through coastal regions (Dergam et al. 2002, Jacobina et al. 2011, Pereira et al. 2013). Indeed, the current distribution of several genera of freshwater fishes such as *Australoheros*, *Cnesterodon*, *Jenynsia* and *Corydoras* in Pampa Plain southernmost areas seems to have been strongly influenced by interconnection of coastal rivers during low sea level periods (Bruno et al. 2011, 2016). Emergence of a coastal plain connecting paleo-basins across Brazil, Uruguay and Argentina coastal regions during these periods would have facilitated spread of freshwater species, occurring several times during at least the last 1-2 millions of years (Ponce et al. 2011).

While glacial – interglacial cycles may have promoted dispersal of *Hoplias malabaricus* coastal populations, at the same time they would also have had profound effects on the Pampa Plain ecosystems. The dominant climate characteristics of the Pampa Plain region during these glacial periods were: cold with frequent snowing and windy conditions, with scarce precipitations and frequent decreases of basin water flow. Thus, cycles of expansion of steppe-like climate over the Pampa Plain region occurred during glacial epochs. Constant propagule pressure from the Brazilian region favored exchange and turnover of species in these regions after glacial periods, and this fauna advance over Pampa Plain region would have taken place approximately 15 times during the last one million years (Rabassa et al. 2005).

The chronological frame of these events fits with the estimated dispersal time of the haplogroup including *Hoplias malabaricus* populations from Ctalamochita and Uruguay Rivers and its tributaries (Santos 2013), thus this climate component of glacial-interglacial cycles must have been a powerful modelling force causing a profound impact over the present geographic distribution of *Hoplias malabaricus* populations living in the Argentinean Central Region.

In light of the previously mentioned data, it is evident that the current geographic distribution pattern of *Hoplias malabaricus* in the Ctalamochita River, and probably most of the ichthyofauna in the Pampa Plain region, is a result of the intricate combination of climatic and geomorphological factors. Overall, cytogenetic and molecular evidence presented in this study highlights the distinctive chromosomal features of *Hoplias malabaricus* from the Ctalamochita River, and also reveals a complex history of dispersal of these populations.

## Conclusion

Taking into consideration the new findings described previously, the present work is the first to provide cytogenetic information and also including some phylogeographic aspects of *Hoplias malabaricus* populations living in close proximity to the southernmost extreme of its distribution area, in Argentinean Central Region. Therefore, this study expands significantly upon the previously known geographical coverage for karyomorph A and contributes to a better understanding of the karyotypic diversification within this species complex.
